# Supplementary material for: The Experiences and Views on Palliative Care of Older People with Multimorbidities, Their Family Caregivers and Professionals in a Spanish Hospital
Source: Healthcare (Basel). 2022 Dec 9;10(12):2489. doi: 10.3390/healthcare10122489 (PMC9778218; doi:10.3390/healthcare10122489)
Supplement: Supplementary file 1 [file healthcare-10-02489-s001.zip › healthcare-1993476-supplementary.pdf]

healthcare

MDPI

**Consolidated criteria for reporting qualitative studies (COREQ): 32-item checklist.**

| No | Item | Guide questions/description |
|---|---|---|
| Domain 1: Research team and reflexivity | | |
| Personal Characteristics | | |
| 1. | Interviewer/facilitator | Laura Llop-Medina (LLL)/Ascensión Doñate-Martinez (ADM) |
| 2. | Credentials | PhD candidate/PhD |
| 3. | Occupation | Researchers at the University of Valencia. |
| 4. | Gender | The researchers were female. |
| 5. | Experience and training | The researchers were trained in qualitative research techniques and had worked on similar studies in the past. |
| Relationship with participants | | |
| 6. | Relationship established | No relationship was established between the participants and the researcher prior to the start of the study. |
| 7. | Participant knowledge of the interviewer | Participants were provided with an information sheet with the purpose of the study and the researcher's credentials. |
| 8. | Interviewer characteristics | Interviewer name, professional affiliation and education were provided. |
| Domain 2: study design | | |
| Theoretical framework | | |
| 9. | Methodological orientation and Theory | Thematic framework analysis, an inherently comparative form of thematic analysis that employs an organised structure of inductively and deductively derived themes (i.e. a framework) to conduct a cross-sectional analysis through a combination of description and abstraction of data. |
| Participant selection | | |
| 10. | Sampling | Participants were purposively selected meeting the criteria of age (older patients) with a diagnosis of complex chronic patients, excluding cancer, and cared for in the home hospitalisation unit of |

| No | Item | Guide questions/description |
| --- | --- | --- |
| | | La Fe hospital. The caregivers had to be caring for a complex chronically ill older patient cared by the home hospitalisation unit of La Fe hospital. The professionals had to be providing direct care to palliative care/chronic patients or managing palliative care services. |
| 11. | Method of approach | Participants were initially identified by a member of the hospital's clinical team, who arranged the interview with the researcher by telephone. |
| 12. | Sample size | 20 participants were interviewed (12 patients, 11 caregivers) and 16 health professionals participated in the Focus Groups. |
| 13. | Non-participation | 10 patients refused to participate due to their delicate health status or the worsening of some of their symptoms, which in some cases led to hospital admission. 3 medical professionals refused to participate due to scheduling problems. |
| Setting | | |
| 14. | Setting of data collection | All the individual interviews were conducted face to face at patient's home. In the case of health professionals, the focus groups took place in a meeting room of La Fe Hospital in Valencia. |
| 15. | Presence of non-participants | There was no one else present besides the participants and researchers. |
| 16. | Description of sample | See Results section. |
| Data collection | | |
| 17. | Interview guide | Interview scripts were provided by the authors, and a pilot test was conducted with a patient and a caregiver to ensure that the questions were understandable. |
| 18. | Repeat interviews | No. |
| 19. | Audio/visual recording | Data were collected via voice recorder after receiving explicit consent from participants. |
| 20. | Field notes | Field notes were taken during the interviews to assist the researchers in the interpretation of the data collected. |
| 21. | Duration | Focus groups: average duration 95 minutes. Interviews: average duration 40 minutes. |
| 22. | Data saturation | Data saturation was discussed and agreed between the research authors of the manuscript. |

| No | Item | Guide questions/description |
|---|---|---|
| 23. | Transcripts returned | Transcripts were not provided but the results were discussed with the participants. |
| Domain 3: analysis and findingsz | | |
| Data analysis | | |
| 24. | Number of data coders | The data were coded by two of the authors of the manuscript (LLL and ADM) |
| 25. | Description of the coding tree | See table 1 |
| 26. | Derivation of themes | Themes were identified as derived from data. |
| 27. | Software | Atlas.Ti software was used to manage the data. |
| 28. | Participant checking | The results were discussed with the participants. |
| Reporting | | |
| 29. | Quotations presented | Quotes from participants were presented to illustrate the main findings. Each quote was identified with the number and profile of each participant, e.g. HP11 (MD specialist in Family and Community Medicine) |
| 30. | Data and findings consistent | The manuscript presents consistency between the data collected and the results described. |
| 31. | Clarity of major themes | The four main themes were presented in the findings in sections |
| 32. | Clarity of minor themes | The 15 sub thematic themes were presented in the section of results. |
